# Speleological and environmental history of Lida Ajer cave, western Sumatra

**DOI:** 10.1098/rstb.2020.0494

**Published:** 2022-04-25

**Authors:** Julien Louys, Mathieu Duval, Gilbert J. Price, Kira Westaway, Yahdi Zaim, Yan Rizal, Aswan, Mika Puspaningrum, Agus Trihascaryo, Sebastian F. M. Breitenbach, Ola Kwiecien, Yanjun Cai, Penny Higgins, Paul C. H. Albers, John de Vos, Patrick Roberts

**Affiliations:** ^1^ Australian Research Centre for Human Evolution, Griffith University, Brisbane 4111 Australia; ^2^ Centro Nacional de Investigación sobre la Evolución Humana (CENIEH), Burgos 09002, Spain; ^3^ School of Earth and Environmental Sciences, The University of Queensland, St Lucia QLD 4072, Brisbane, Australia; ^4^ School of Social Science, The University of Queensland, St Lucia QLD 4072, Brisbane, Australia; ^5^ Department of Earth and Environmental Sciences, Macquarie University, Sydney, New South Wales, Australia; ^6^ Geology Study Program, Institut Teknologi Bandung, Jawa Barat 40132, Indonesia; ^7^ Department of Geography and Environmental Sciences, Northumbria University, Newcastle upon Tyne, UK; ^8^ Institute of Global Environmental Change, Xi'an Jiaotong University, People's Republic of China; ^9^ EPOCH Isotopes, 6606 E Townline Road, Williamson, NY 14589, USA; ^10^ Naturalis Biodiversity Center, Leiden, The Netherlands; ^11^ Max Planck Institute for the Science of Human History, Kahlaische Strasse 10, 07745 Jena, Germany

**Keywords:** early humans, Pleistocene, rainforest, Southeast Asia, fossils

## Abstract

Some of the earliest evidence for the presence of modern humans in rainforests has come from the fossil deposits of Lida Ajer in Sumatra. Two human teeth from this cave were estimated to be 73–63 thousand years old, which is significantly older than some estimates of modern human migration out of Africa based on genetic data. The deposits were interpreted as being associated with a rainforest environment based largely on the presence of abundant orangutan fossils. As well as the main fossil-bearing chamber, fossil-bearing passages are present below a sinkhole, although the relationship between the different fossil deposits has only been tenuously established. Here, we provide significant new sedimentological, geochronological and palaeoecological data aimed at reconstructing the speleological and environmental history of the cave and the clastic and fossil deposits therein. Our data suggest that the Lida Ajer fossils were deposited during Marine Isotope Stage 4, with fossils from the lower passages older than the main fossil chamber. Our use of stable carbon and oxygen isotope analyses of mammalian tooth enamel demonstrates that early humans probably occupied a closed-canopy forest very similar to those present in the region today, although the fossil orangutans may have occupied a slightly different niche.

This article is part of the theme issue ‘Tropical forests in the deep human past’.

## 1. Background

Understanding how and when *Homo sapiens* expanded out of Africa and into Europe and Asia has been the subject of much recent debate. Genomic and mitochondrial data have frequently been used to argue for a major exit from Africa around 65–60 thousand years ago (e.g. [1,2]). However, archaeological evidence from Saudi Arabia, Israel, Greece and China indicates that at least some populations occupied Europe and Asia before this time [3–7], most likely following corridors of suitable habitats resulting from ameliorated climatic conditions [8,9]. The Sumatran palaeontological record, especially the remains recovered from the site of Lida Ajer in the Padang Highlands, plays an important role in these debates owing to both its age and proposed environmental context [10].

In the late 1880s, Eugene Dubois travelled to Padang, Sumatra in search of the missing link between humans and other apes. He explored many caves in the region, and thousands of fossils were extracted under his direction [11]. Recovering only modern mammals, Dubois became convinced that the deposits were Holocene in age, and he abandoned them in favour of excavations in Java in 1890. Nevertheless, the wealth of fossils recovered lent themselves to further examination. Research on the Sumatran fossils collected by Dubois continued sporadically through the twentieth century (e.g. [12,13]). Close study of the orangutan fossils from Lida Ajer by Hooijer [14] revealed that two of the hominid teeth recovered were in fact modern human. While the Sumatran caves preserved only modern species, the close taxonomic and depositional relationships between the Lida Ajer and Sibrambang caves in Sumatra, and the site of Punung in Java, suggested to de Vos that these sites could be contemporaneous and Late Pleistocene in age [13]. Establishing an age of approximately 118 ka (kilo annum BP) for Punung [15] suggested that the Sumatran caves may be of similar antiquity (e.g. [16]).

**Figure 1.**
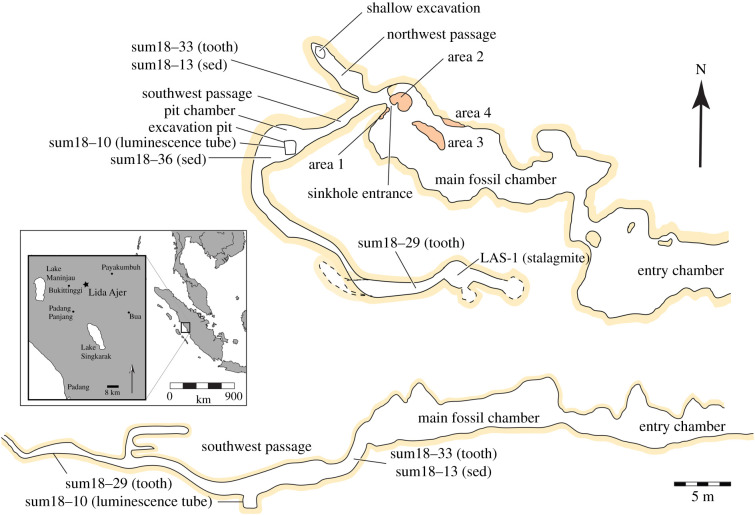
Map of Lida Ajer cave showing the sinkhole in relation to the main fossil chamber described by Dubois [17] and Westaway *et al*. [10]. Plan and profile views of the cave shown, with major finds discussed in the text indicated. Inset: Location of Lida Ajer shown with respect to major geographical features in the region and Sumatra. (Online version in colour.)

A reinvestigation of Dubois's Sumatran caves began in the 1990s, with particular attention focused on Lida Ajer, and a comprehensive dating programme of this cave was initiated in the late 2000s. Luminescence and uranium-series (US) dating applied to sediments and associated speleothems in the main fossil-bearing chamber (figure 1), combined with US and electron spin resonance (ESR) dating of mammalian teeth recovered by Dubois and from new excavations, respectively, revealed that the deposits were at least 63 ka [10]. Largely because of the presence of orangutans in the deposits of Lida Ajer, the site was taken to represent rainforest habitats [10,13]. A reinvestigation of the human teeth also confirmed their taxonomic identity [10]. These findings had two major implications. First, they corroborated the notion that humans left Africa significantly earlier than commonly cited estimates based on genetic evidence that placed human migration out of Africa at approximately 60 ka [1,2]. Second, because the fauna from Lida Ajer was considered typical of contemporary rainforest environments [10,13], these results were argued to represent some of the earliest evidence of human presence in rainforests globally.

Dubois's initial investigations at Lida Ajer focused on the main fossil chamber that was also the subject of the integrated dating programme discussed above. However, as work continued at the site, Dubois's team began enlarging a hole towards the back of this chamber, work which necessitated removal of large sections of overlying breccia [17]. This hole, dubbed the Lida Ajer sinkhole [10], connects the main fossil-bearing chamber to two additional passageways to the west, both also yielding fossils. These deposits were interpreted, based on field observations, as washed in material eroded from the main fossil-bearing chamber [10]. Although these passages are briefly discussed in Dubois's field reports and Westaway *et al*. [10], they remain to be fully described, and the relationship between fossils in the sinkhole and the main fossil deposits have not been firmly established. This is important because the Dubois fossil material now housed in the Naturalis Biodiversity Centre (Leiden, The Netherlands) does not have specific *in situ* data associated with individual finds. Thus, exact locations of individual fossils from Dubois's collection, including the human remains, could be derived from either the main fossil chamber or the sinkhole.

Here, we provide a description of these passageways and detail new geochronological, sedimentological, and historical data to establish the relationship between the sinkhole deposits and the main fossil chamber. The exact nature of the palaeoenvironments that humans from Lida Ajer inhabited also remains to be established. How Pleistocene rainforests in the Padang Highlands may have differed from modern rainforests has not been explored but is critical in understanding the nature of the long-term human occupation of these ecosystems. Moreover, the use of orangutans as a palaeohabitat indicator for rainforests, as assumed for Lida Ajer, may not be straightforward as these species may have broader ecological tolerances than previously appreciated [18]. To explore these issues further, we reconstruct the palaeoenvironmental context of the Lida Ajer deposits using stable carbon and oxygen isotope analysis of mammalian tooth enamel from both recently collected fossils and historical fossils extracted from the cave by Dubois's team. These are compared to existing data for modern Southeast Asian species and previous palaeoenvironmental reconstructions for the site.

## 2. Material and methods

### (a) Speleology and sedimentary infill of the cave

The two front chambers of Lida Ajer were described by Westaway *et al*. [10] and briefly summarized here. The easterly cave entrance measures 4.8 m wide by 2.1 m high and is situated in a limestone hill above the Batang Babuwe River. Limestone deposits continue above the cave although their exact height is obscured by dense vegetation. The cave opens into a moderately sized circular chamber with speleothem decorations and columns (figure 1). The floor is unconsolidated sediment which shows signs of anthropogenic modification (digging and overturning). A second chamber is accessed through a narrow opening between speleothem formations. It opens into a longer more cylindrical chamber with additional speleothem formations at the eastern end. The western end of this chamber is dominated by stalactite columns hanging from the centre of the roof (area 3 in [10]). Immediately below these and along the walls at the back of the chamber (areas 1 and 4) are cemented breccia deposits. A large breccia deposit with significant overlying flowstone (area 2) is observed at the northwestern end of the chamber.

In 2018, we surveyed and mapped the sinkhole passages in Lida Ajer using traditional cave survey techniques and a laser range finder. We conducted spot collection of fossils and geological samples along the passages. These consisted of a broken stalagmite (LAS-1), two micromorphology sediment samples (results to be reported elsewhere), and sediment samples and/or teeth for luminescence and ESR dating (figure 1). Analysis of the stalagmite is detailed in the electronic supplementary material. Where evident, we noted sedimentary information associated with exposures. These were combined with the field report from [17] to reconstruct the likely geological condition of the cave prior to excavation, and to determine the speleological history of the fossil-bearing and associated deposits.

### (b) Geochronology

US dating of four samples (LAS-A-U1 and LAS-1-U2-4) taken from stalagmite LAS-1 was performed on a ThermoFisher Neptune multicollector-inductively coupled plasma-mass spectrometer at the isotope laboratory at Xi'an Jiaotong University, Shaanxi Province, China (see the electronic supplementary material). Two fossil teeth were collected from the sinkhole at Lida Ajer for dating: one rhinoceros tooth (SUM18-29) and one orangutan tooth (SUM18-33_t_). Three sediment samples were collected for the corresponding dose rate evaluation (details in the electronic supplementary material). The teeth were prepared and dated by means of US and combined US/ESR dating methods (see [19] for basic principles) following the same procedure employed on teeth from other Sumatran sites [20,21]. ESR dose evaluations were performed on enamel powder at CENIEH, Spain, using the multiple aliquot additive dose (MAAD) method. Detailed ESR data are displayed in the electronic supplementary material, figure S4 and table S2. Solution US analyses of powdered dental tissues were carried out at the Radiogenic Isotope Facility of The University of Queensland, Australia. US isotopic data are displayed in the electronic supplementary material, table S3. Combined US-ESR dating was performed using the DATA program [22]. Full details on the analytical procedure, including sample preparation, *D*_E_ calculation and dose rate evaluation, are provided in the electronic supplementary material. US-ESR data inputs and outputs are given in the electronic supplementary material, table S5. An opaque tube of sediments was collected from the clay-silt bed at the base of the pit in the sinkhole for post-infrared stimulated luminescence (pIR-IRSL) dating (SUM18-10). The sediments were prepared and dated using single-grain pIR-IRSL techniques as employed in other caves in the region [21], and the environmental dose rate was estimated using a combination of alpha and beta counting. Full details on the sample preparation, *D*_E_ and dose rate evaluation are provided in the electronic supplementary material. ESR and IRSL age results are given a 1*σ* confidence level unless otherwise indicated.

### (c) Stable isotope analysis

Twenty-eight teeth were sampled for stable carbon (*δ*^13^C) and oxygen (*δ*^18^O) isotope measurement, 20 from the Dubois collections of the Naturalis Biodiversity Centre and eight from the Institute of Technology, Bandung (ITB). This approach has been demonstrated to provide important insights into Pleistocene faunal (including hominin) feeding behaviours and broader palaeoenvironmental conditions (e.g. water availability) in the tropics of Asia [23–25]. Dubois's samples were taken from fragmented material without individual accession numbers (bulk accession numbers listed in the electronic supplementary material); they are referred to here as Lxxx-X, with these identities noted with the specimens and left in the collection. The 20 tooth fragments sampled from Naturalis include two Bovidae (*Capricornis sumatraensis*), seven Elephantidae (*Elephas maximus*), one Hominidae (*Pongo* sp.), nine Rhinocerotidae (gen. *et* sp. indet.), and one Tapiridae (*Tapirus indicus*). Because of the fragmented nature of the teeth, we cannot strictly exclude the possibility that we sampled the same individual within the taxonomic groups. Eight whole and partial teeth were selected from the ITB collections (numbered LAxx-x here): two Hominidae (*Pongo* sp.), two Rhinocerotidae (?*Rhinoceros* and ?*Dicerorhinus*) and four Suidae (*Sus* sp.). Of these, four were derived from the sinkhole and four from the main fossil chamber (electronic supplementary material, table S7). Sampling, pre-treatment and analytical procedures are detailed in the electronic supplementary material, with all *δ*^13^C values reported as dietary *δ*^13^C.

Stable isotope values were compared to modern baseline taxa, largely derived from Louys & Roberts [25] but supplemented by modern and Holocene taxa presented by Janssen *et al*. [26], Puspaningrum *et al*. [27] and Bocherens *et al*. [28] (for complete dataset see supplement in [25]). Comparisons between modern and fossil taxa were threefold: with modern Southeast Asian species but restricted to the families sampled from Lida Ajer (case 1); with all modern species with provenance data from Sumatra (case 2); and modern Sumatran species restricted to the families sampled from Lida Ajer (case 3). These were facilitated by Mann-Whitney pairwise comparisons and kernel density plots, with analyses computed in PAST v. 2.17c [29].

## 3. Results

### (a) Speleology and sedimentary infill of the cave

**Figure 2.**
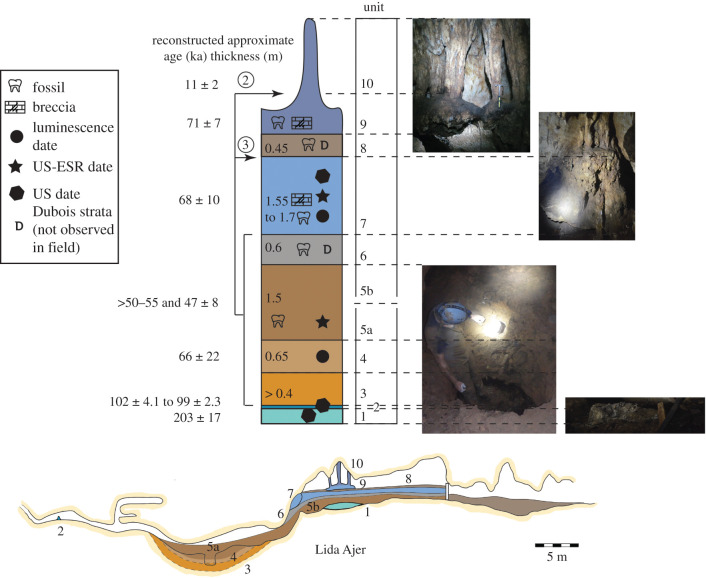
Reconstructed speleological history of Lida Ajer showing the likely cave deposits present when Dubois first entered the cave. Units 1 to 10 are shown in profile view and in our preferred chronostratigraphic sequence. Observed and reconstructed thickness of each clastic unit indicated in metres. Alternative sequences are shown with circled numbers indicating where the bracketed units would move to in each alternative chronostratigraphic scenario. All ages are reported at 2*σ*. (Online version in colour.)

The oldest deposits recorded at Lida Ajer are the basal flowstones in the main fossil chamber. These were dated to 203 ± 17 ka (2*σ*) [10] and we have correspondingly referred to these as unit 1 (figure 2). Dubois reported several sedimentological units in this chamber [17]. The lowest was a ‘brownish clay’ that preserved teeth and bones which we call unit 5b (units 2–5a are located deeper in the cave and discussed below). Above this, Dubois records a sandy unit of 60 cm thickness that he speculates includes ‘pumice-tuff’ inclusions which we call unit 6. Above this layer, Dubois reports an enormous ‘stalagmite breccia’ that extended across the floor of the cave and up to 1.7 m thick and that we correlate with areas 1–4 of Westaway *et al*. [10]. We refer to this breccia as unit 7. Units 5b, 6 and 7 were reported by Dubois to host fossils and were interpreted as being redeposited in mass flow events by Westaway *et al*. [10] after initial bone accumulation by porcupines. Above these units, Dubois reports ‘layers of soil glued with chalk’ of 45 cm depth which we have called unit 8. Dubois indicates that above the soils sat the flowstones, US dated by Westaway *et al*. [10] to 71 ± 7 ka (2*σ*), which are here designated unit 9. Finally, the hanging stalactite columns, also dated (11 ± 2 ka, 2*σ*), are designated as unit 10.

The sinkhole entrance is situated between and slightly under areas 1 and 2 (figure 1). Dubois [17] notes the sinkhole opening at the back of the main fossil chamber ‘was completely filled with earth’ when he began extending his excavations. Today it has a steep decline for approximately 1.2 m before levelling out and two passages continue at its base, one short passage oriented northwest and a second southwest. The walls in the passages are smeared in unconsolidated muds with numerous limestone clasts and fossils exposed. The northwest passage is ca. 1.5 m wide and extends for almost 7 m. A visual connection can be established with the main chamber in an easterly direction at the beginning of this passage in what appears to be an excavated niche, north of area 2. However, physical passage was not possible. There is a shallow excavation pit visible at the end of the northwest passage.

The southwest passage extends for approximately 7 m before opening into a wider (2.6 × 7.2 m) ovate chamber. This almost certainly corresponds to the ‘room’ discussed by Dubois [17], which he described as almost filled to the ceiling, with oxygen levels so depleted that candles would not burn. The chamber is at least 1.5 to 2 m high today, suggesting a significant amount of sediment had been removed by Dubois's team. This was further corroborated by the discovery of a square pit near the entrance to this chamber measuring 1.3 × 0.9 m in area and 1.1 m deep. The base layer of this pit consists of ca. 40 cm of massive clays which we designate as unit 3. Overlying this is a 72 cm bed of clays with clay-silt and quartz clasts with occasional horizontal bedding visible and limestone clasts (unit 4). Neither of these beds contain any fossils, and the luminescence dating sample was taken from unit 4.

Above unit 4 is a *ca* 38 cm thick clay and sandy-silt layer. This bed had very fine laminations and contained numerous very coarse quartz and volcanic angular clasts. Fossils were found only on the very top of this layer but not observed in the pit walls. We think it is highly likely that the remnants of this unit are the clays and muds observed on the walls, floor, and roof of the sinkhole passage and that prior to Dubois’ excavations these would have filled most of the passageways. We designate these fossil-rich clays and muds as unit 5a. As Dubois's report [17] mentions that this chamber was ‘almost filled up to the ceiling’, an additional 1 m can be added to the unit thickness.

Given that the sinkhole entrance was filled, we also think it is reasonable that the unit 5a stratum extended into the main fossil chamber and is the same as Dubois's ‘brownish clay’ that we designated unit 5b. Our observations in the cave as well as Dubois' notes strongly suggest that a continuous unit (unit 5) extended from the pit chamber through the passages east of the pit chamber (unit 5a), largely filled the sinkhole and continued into the main chamber (unit 5b). However, the apparent complete removal of unit 5b from the main fossil chamber by Dubois makes it impossible to correlate these two depositional units unambiguously. The laminated sand-silts at the base of unit 5 alongside the presence of coarse clasts suggests deposition in alternating low and high energy water although any potential changes in sediment flow at the top of this unit are not preserved.

The passage exiting the pit chamber proceeded in a southeast direction and became very narrow such that it was difficult to traverse. Walls, floor and roof were composed of unconsolidated muds in which numerous fossils were collected, and these are also correlated with unit 5. Approximately 12–15 m into this passage the roof opened vertically, and two false floors made of flowstone carbonate and extending back northwest were recorded. These were relatively free of the muds and contained no visible fossils. The main passageway extended beyond these false floors to another small circular chamber, with at least two additional passages extending from it. These were not explored further owing to the difficulty of access. Just west of this small circular chamber the broken stalagmite LAS-1 was recovered from the muds that we attribute to unit 5b. Its location and preservation suggest it was only, recently, broken and is allochthonous to this part of the cave, and we refer to its speleothem deposit as unit 2. As it was found within the muds, unit 2 is older than unit 5a.

### (b) Geochronology

#### (i) Age and growth rate of the stalagmite

The US dating (electronic supplementary material, table S1) revealed that LAS-1 grew between 102 ± 4.1 ka and 99 ± 2.3 ka and may be correlated to Marine Isotope Stage 5c (MIS 5c; *ca* 108–93 ka, [30]). The high uncertainty stems from the high content of detrital thorium which limits the accuracy of our radiometric chronology and hampers comparisons with existing palaeoclimate records from the region. A greyscale-based layer-counted floating chronology (see the electronic supplementary material for details) suggests that the stalagmite grew for 586 ± 14 years (electronic supplementary material, figure S2). Growth rate, represented by annual layer thickness, varies from 43 µm a^−1^ to 650 µm a^−1^, with a median of 151 µm a^−1^. The grey values vary from 100 to 240 (figure 1*b*). There is no apparent correlation between the pattern of grey values and layer thickness, but individual grey value peaks generally correspond to thicker layers in more porous intervals (electronic supplementary material, figure S3). There is a visual correlation between grey values and the presence of brown-to-orange layers, with lower values (darker colour) corresponding to intervals with high frequency of brown-to-orange layers.

#### (ii) Solution uranium-series analyses of dental tissues

Solution US analyses of the bulk powdered dental tissues returned apparent age estimates ranging from *ca* 37 to 58 ka (electronic supplementary material, table S3). No significant detrital thorium contamination was observed in the samples, resulting in very limited corrections of the apparent ages (less than 3 ka). Because all samples return finite US estimates there was no immediate evidence of uranium leaching. Moreover, the fact that the dated specimens generally have very low uranium concentrations (less than 0.5 ppm), including for the enamel, made them suitable for ESR dating. The apparent US age estimates should be regarded as minimum age constraints for the fossils, and are consistent with those of the associated luminescence ages (i.e. they are generally younger than the burial ages).

#### (iii) Combined uranium-series and electron spin resonance dating of fossil tooth enamel

The three samples of raw sediment collected for dosimetric purposes returned highly variable results (between 27 and 68%) for each element (electronic supplementary material, table S4). Because tooth SUM18-29 had no directly associated (or attached) sediment available, combined US/ESR age calculations were first successively performed using each of the three sediment samples for the beta and gamma dose rate evaluation (see data inputs in the electronic supplementary material, table S5). None of the calculations returned a finite age result. Under normal circumstances, this would indicate that all dental tissues have experienced uranium leaching. However, given the significant uncertainty around the dose rate evaluation, such an interpretation is treated with caution. We cannot exclude that the true external beta and gamma dose rate values are much lower than those calculated from the sediment sample, and the presence of the cave walls within a short distance of the sample could significantly lower the dose rate (e.g. [20]). Consequently, combined US/ESR dating of this tooth does not provide any conclusive chronological result and cannot be used to demonstrate uranium leaching. Evidence indicates that the apparent US age estimates can be considered reliable age constraints, with dental tissues suggesting a minimum age of 50–55 ka for SUM18-29.

Two sediment samples were more closely associated with tooth SUM18-33_t_. Sample SUM18-13 was taken *in situ* from the area around the tooth, while sample SUM18-33_s_ was attached to the tooth and collected during sample preparation. Dose rate calculations were first performed using sediment sample SUM18-33_s_ for both beta and gamma dose rate components, resulting in a US-ESR age of 47 ± 4 ka (electronic supplementary material, table S5). Calculated *p*-values show early uptakes in both tissues (−0.96 < *p* < −0.73). The corresponding closed system US-ESR age estimate is only 1 ka older, showing the negligible impact of uranium uptake modelling on age results. When deriving a gamma dose rate value from the mean radioelement concentrations of samples SUM18-13 and SUM18-33_s_, a younger US-ESR age of approximately 40 ka may be obtained. In the absence of *in situ* measurements of radioactivity, the gamma dose rate evaluation is very challenging: we cannot reasonably exclude that the cave wall may significantly contribute to the gamma dose rate, and thus make the resulting age estimate older. If so, this radioactively almost inert material would most likely contribute to lower the true gamma dose rate. An extended discussion of the combined US-ESR age results may be found in the electronic supplementary material. Because this uncertainty cannot be quantified at present, it is reasonable to consider the calculated US-ESR age estimates as minimum ages, although the magnitude of this potential underestimation is unknown.

#### (iv) Luminescence dating

The luminescence characteristics of the feldspars from the sinkhole (electronic supplementary material, figure S5) were as consistent as those seen in the main chamber [10]. The low feldspar yield, which is a feature of the cave sediments from this region (e.g. [21]; [10]), resulted in only 20 feldspar being accepted. This is not a statistically significant number but the use of single-grains rather than the single aliquots used to constrain the main chamber [10] provides a useful assessment of the grains that have been the most bleached before being buried in the cave, and a close estimation of the burial age. This result (66 ± 22 ka) (electronic supplementary material, table S6) is apparently slightly older than the breccia from the main fossil chamber (62 ± 5 ka) published by Westaway *et al*. [10]. Such close burial age estimates may indirectly suggest a relatively rapid sedimentation process in the cave from unit 4 to unit 7. However, the apparent age difference may also not be significant given the relatively large associated age error (*ca* 33%) from the lowermost sample. This age estimate carries significant uncertainty on the external dose rate owing to the strong heterogeneity of the breccia deposits and the absence of *in situ* gamma dose rate.

### (c) Stable isotope analysis

**Figure 3.**
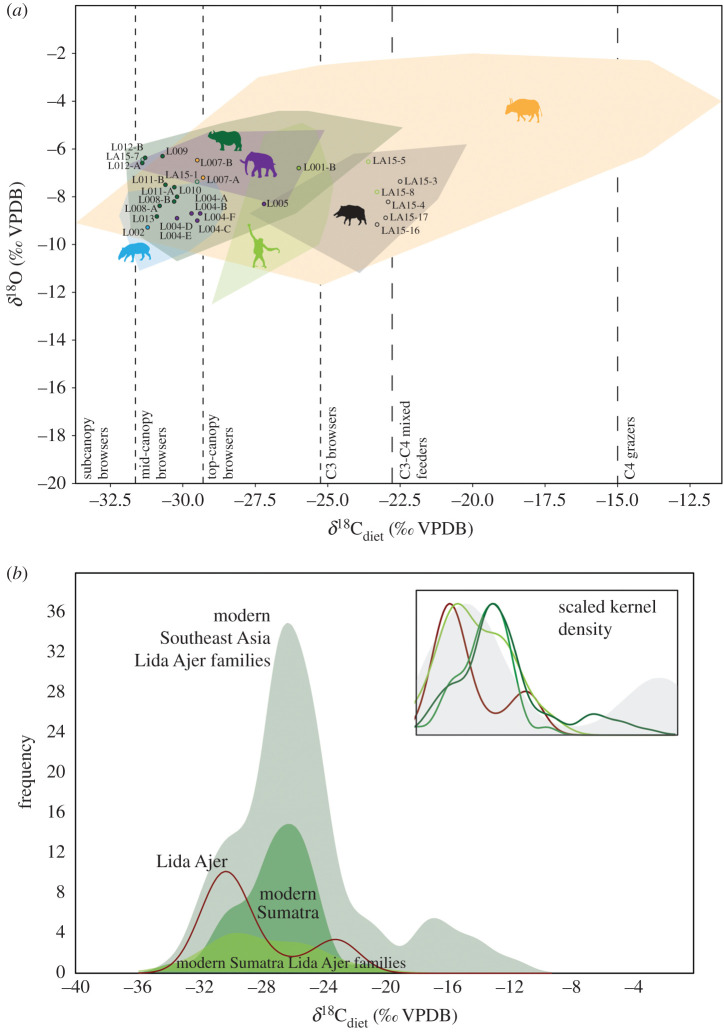
Stable isotope analysis of *δ*^13^C_diet_ (‰ VPDB) and *δ*^18^O (‰ VPDB) from faunal enamel of mammals from Lida Ajer. (*a*) Comparison of Lida Ajer specimens (closed circles indicate fossils sampled directly from Lida Ajer; open circles represent fossils sampled from the Naturalis) against all Southeast Asian representatives from those families (convex hulls). Divisions between canopy browsers indicative and based on Louys & Roberts [25]. Animal silhouettes from Phylopic.org (public domain and CC BY-SA 3.0 CC BY 3.0 *Pongo* by Gareth Monger and CC BY-NC 3.0 Elephantini by Zimices). (*b*) Kernel density plots for Lida Ajer and modern subsets of Southeast Asian mammals. Inset: kernel densities artificially scaled to the same frequency and compared against a subtropical monsoon forest in China; drawn after Tejada *et al*. [31] using data from Ehleringer *et al*. [32]. (Online version in colour.)

*δ*^13^C and *δ*^18^O values for the fossil samples are provided in the electronic supplementary material, table S7. While sample sizes are too small to quantitatively compare the samples from the sinkhole with those of the main fossil chamber, qualitatively they are very similar: a *δ*^13^C difference of 0.4‰, 1.9‰ and 0.5‰ for orangutans, rhinos and pigs, respectively (difference in mean sinkhole versus mean main chamber for pigs); and 1.3‰, 0.9‰ and mean 1.2‰ for *δ*^18^O. The mean *δ*^13^C for the Lida Ajer fossils is -28.5‰, well within values considered typical of closed-canopy rainforests (figure 3*a*), with the highest *δ*^13^C value (from a pig) still within the range of C_3_-dominated ecosystems.

Comparison of the *δ*^13^C of the Lida Ajer data with the available modern Southeast Asian dataset reduced to families available at Lida Ajer (case 1) shows statistically significant differences (*U* = 956.5, *p* < 0.001), with the Lida Ajer sampled community recording more negative *δ*^13^C values. This can be most readily seen in the kernel density plot (figure 3*b*), with the modern Southeast Asian families having a long right tail representing the small but significant C_4_ grazing species in the Bovidae. This is also evident in the right side of the plot in figure 3*a*. In addition, two other taxa from Lida Ajer show isotope values outside the range of modern families. Small sample sizes prevent quantitative examination. However, two of the three Lida Ajer orangutans have more positive *δ*^13^C values than any of the modern species sampled, and all the elephants have more negative *δ*^18^O values than modern specimens (figure 3*b*). At a community level, however, there are no significant differences in *δ*^18^O between Lida Ajer and modern Southeast Asian species (*U* = 1273, *p* = 0.14).

Restricting comparisons to only Sumatra, when the Lida Ajer fossils are compared to modern Sumatran species across all families with *δ*^13^C data available (case 2), the Lida Ajer sampled community is still significantly lower (*U* = 376, *p* = 0.02). This is demonstrated in the higher peak in values observed in the kernel density plot (figure 3*b*). However, examining the modern Sumatran data reduced to the families available at Lida Ajer (*U* = 198, *p* = 0.35) produces no significant *δ*^13^C differences (figure 3*b*). As a result, while the community-level comparisons suggest strong differences between modern forest faunas and those present in the Late Pleistocene in Sumatra, this seems to only be owing to the wider taxonomic sampling of the modern Southeast Asian and Sumatran datasets compared to the taxa sampled from Lida Ajer. A more reasonable comparison between families found in Lida Ajer to those present in Sumatra shows no significant community-level differences.

## 4. Discussion

### (a) Speleological history

The speleological history of the site is summarized in figure 2. During the deposition of unit 1 at *ca* 200 ka, the main fossil chamber experienced vadose conditions. We have no cave records for another approximately 100 ka. This may be owing to several factors: (i) insufficient cave exploration and sampling of speleothems (sampling bias); (ii) the cave was flooded or filled with sediment during this time (in both cases hindering speleothem formation); and/or (iii) speleothems did continue to grow but were subsequently broken and/or displaced by high energy flood events. The broken nature of stalagmite LAS-1 testifies to the probability of the last factor at work. Testing these hypotheses will require more targeted sampling and analysis of speleothems, especially in the sinkhole. Between 102 and 99 ka, vadose conditions are generally present in at least the sinkhole, as evidenced by the formation of stalagmite LAS-1 (unit 2), although frequent flooding of at least these lower passages is recorded in its layers (electronic supplementary material). Clastic sedimentation post-dates the formation of the stalagmite. The numerical ages (and their associated uncertainty) produced for each of the sedimentary layers and/or their fossils allow for different interpretations regarding the sequence of deposition. The most parsimonious interpretation, scenario 1, assumes conventional stratigraphic order following the law of superposition. Two alternative scenarios incorporating reverse stratigraphy are also discussed. All numerical age results reported hereafter are given at 2*σ*.

In scenario 1, we assume that the massive clays (unit 3) at the base of the sinkhole pit represent the oldest clastic deposits in Lida Ajer. The clays suggest deposition from standing water. This unit is directly overlain by more silty clays (unit 4) that indicate some limited water movement. A luminescence date for unit 4 suggests sediment deposition between 88 and 44 ka. Neither of these units appears to preserve fossils. Unit 4 is overlain by laminated sandy-silts and clays with numerous clasts (unit 5), with the top of this unit preserving fossils. Two teeth from this unit, one near the sinkhole entrance and the other beyond the excavation pit, have returned ages of 50–55 ka (US) and 47 ± 8 ka (combined US-ESR), both interpreted as minimum age constraints for the fossils. These are consistent with the luminescence dates from the underlying deposits, suggesting a burial age range of 88–55 ka for unit 4.

We found no evidence of Dubois's ‘pumice-tuff’ layer (unit 6) that overlies unit 5 in the main fossil chamber. If indeed this unit preserved pyroclastic sediments, then several large eruptions on Sumatra may have provided the source of the sediments that would have entered the cave as a re-depositional fraction. These include Toba (74.2 ± 0.2 ka; [33]), Maninjau (52 ± 3 ka; [34]) or Masurai (33 ka; [35]) (see also [36]). If our proposed scenario is correct, then the volcanoclastics of unit 6 would be constrained in age between unit 4 (less than 88 ka; 2-sigma lower range of 66 ± 22 ka) and unit 7 (greater than 58 ka; 2-sigma lower range of 68 ± 10 ka; see below), consistent with the Toba eruption (74.2 ± 0.2 ka; [33]).

The enormous ‘stalagmite breccia’ (unit 7) that extended across the floor of the cave probably corresponds to breccia dated by Westaway *et al*. [10]. Modelled ages suggested the breccia formed 68 ± 10 ka. We did not observe unit 8 in the field but above this layer are the unit 9 flowstones dated to 71 ± 7 ka, and finally the hanging columns, unit 10, dating to 11 ± 2 ka [10].

Taking the mean ages from units 4 and 5 suggests the sinkhole deposits are younger than the main fossil chamber deposits (units 7–9). However, considering the 2-σ age errors, unit 4 could be as old as 88 ka while the unit 7 breccia could be as young as 58 ka and the unit 9 capping flowstone in the main fossil chamber as young as 64 ka. Moreover, the apparent stratigraphic inconsistency between the ESR age estimates from the sinkhole and the main chamber may simply result from the non-negligible uncertainty on the gamma dose rate given the highly heterogeneous nature of the sediments in the sinkhole (electronic supplementary material, figure S6) (as well as in the main chamber), and unknown proximity to limestone walls. Indeed, using gamma dose rates from the main fossil chamber as per Westaway *et al*. [10] increases the age of these teeth to 80 + 8–7 ka and 89 + 10–9 ka, consistent with deposition of unit 5 prior to units 7 and 9 (see discussion in the electronic supplementary material).

On balance, the fossils in the sinkhole are most likely older than the unit 7 breccia dated by Westaway *et al*. [10]. Our preferred scenario assumes the correlation of the sinkhole sediments and the main chamber clays described by Dubois is correct, although is not dependent on this. Nevertheless, we cannot exclude alternative scenarios based on the data available. The unit 5 sediments may have provided accurate gamma dose rates for the unit 5 fossil age calculations. If so, and the unit 5 fossil ages represent true ages as opposed to minimum ages, it would require that unit 5 (and potentially units 3 and 4 also) were deposited after the unit 9 and 10 cemented deposits. One alternative scenario (scenario 2) could be that units 7 and 9 formed a false floor under which the unit 5 sediments were deposited after the older deposits were washed out of the cave, possibly by some of the recurrent flooding events evidenced in the cave from the study of the stalagmite LAS-1. Following deposition of unit 5, unit 10 would form. However, we consider this unlikely because it would require the suspension of up to 2 m of breccia in the main fossil chamber without any columnar anchoring, or alternatively no connection between the sediments in the sinkhole and the main fossil chamber.

A second alternative (scenario 3), combining the gamma dose rates from the main fossil chamber for the unit 5 fossils and reverse stratigraphy, would require the deposition of unit 5 below unit 7 but prior to the deposition of unit 9. In this scenario, the age of unit 5 would be constrained between 71 ± 7 ka (unit 9) and 68 ± 10 ka (unit 7). Westaway *et al*. [10] originally hypothesized that the sinkhole deposits represented eroded material from the main fossil chamber breccias and washed into the lower passages, a hypothesis consistent with scenario 3 and some of the features observed in the fossil teeth (see the electronic supplementary material). We now consider this unlikely owing to the very large amounts of sediment required to fill the sinkhole to the roof as observed by Dubois—these would have had to both come from the main fossil chamber and refill it.

### (b) Palaeoenvironmental history

Regardless of deposition scenario preferred the fossils from Lida Ajer most likely date to MIS 4 (76–59 ka, using the composite marine *δ*^18^O record provided by [37]), as the fossil-bearing units have ages of greater than approximately 50 ka (unit 5) and 68 ± 10 ka (unit 7) and 71 ± 7 ka (units 8 and 9). The stable isotope results unambiguously indicate that the mammals of this time occupied a largely closed-canopy tropical forest environment (*δ*^13^C_diet_ −22.5‰ to −31.4‰, mean −28.5‰) very similar to rainforest ecosystems present in Sumatra today. This supports previous interpretations based on the presence of modern rainforest species, and especially orangutans, at the site [10,13]. Nevertheless, there are indications from the stable isotope data that the division of niche space within these forests was not directly analogous to modern ones. This is most clearly represented by the orangutans and elephants. Two of the orangutans have higher *δ*^13^C values than modern species. The modern sample includes one modern Sumatran orangutan (*Pongo abelii*), suggesting that this is not owing to differences between Bornean and Sumatran species, as has been observed in the microwear signatures of fossil and modern species [38]. Rather, it suggests broader diets in the Lida Ajer orangutans, towards the mixed C_3_-C_4_ part of the spectrum compared to modern taxa and compatible with the idea that Pleistocene orangutans exploited more diverse environments in the Pleistocene [18]. The differences in *δ*^18^O values for the elephants could be a result of ecological or climatic differences. The former may include differences in mobility patterns and behavioural or dietary preferences between modern and fossil elephants. The Lida Ajer elephants may have preferentially targeted fruits or forage in the understory, which have lower *δ*^18^O values [39]. Consuming vegetation from karst forests such as those present around Lida Ajer may also lead to lower *δ*^18^O, as these plants often experience water stress owing to thin soils and rapid drainage [40]. Alternatively, elephants range widely, and it has been suggested that this can make them deviate from local scale changes in *δ*^18^O (e.g. [41]). In this context, differences in *δ*^18^O for elephants could be linked to wetter conditions. Wetter conditions, particularly in monsoonal domains, may result not only from the amount (magnitude), but also the distribution and timing of changes. Consequently, wetter conditions do not necessarily imply an increase in total precipitation but rather can point to prolongation of the wet season [42]. Such changes, recorded in the geochemical signature of tooth enamel, may not be captured in vegetation structure. Resolving between these factors will require regional climatic records spanning the ages of the Lida Ajer elephant remains.

Previous synecological reconstructions of Late Pleistocene Lida Ajer suggested that tree canopy cover was mostly dominated by light cover, with only a minor closed-canopy component [18]. This does not reconcile immediately with the stable isotope data presented above. A somewhat more open component of the canopy is suggested by higher *δ*^13^C values of all the pigs and two of the orangutans. This is also supported by the lack of very low (e.g. < −32‰) *δ*^13^C values in Lida Ajer, which would be expected in rainforest subcanopy browsers exclusively foraging under complete canopy cover [25,31,43]. Nevertheless, the habitat was still clearly heavily forested with strong elements of closed canopy. At 675 m above sea level, Lida Ajer today sits in the upper slopes of characteristic karst hill vegetation, and below the 800 m altitudinal boundary that delineates more open montane vegetation [44]. The lower aspects of karstic hills are characterized by 25–30 m canopies with an occasional emergent tree reaching 55 m, while the ground layer is dominated by rattans and palms [44]. Ridges do break up the canopy cover in the higher sections of karst hills above 500 m, and forest diminishes in size with only a few emergent trees reaching 20 m and with most trees only reaching 5–10 m in height. Gaps in relative cover reach in the order of approximately 10% of the surface [44].

The more open environmental reconstruction for Lida Ajer calculated by Spehar *et al*. [18] is therefore probably influenced by the presence of forest bovids in Sumatra, e.g. *Capricornis sumatraensis,* that in our isotopic analyses have very low *δ*^13^C values, but which can otherwise be more open-adapted in the broader Southeast Asian region (right section of figure 3*a*). Modern sampled taxa from Southeast Asia, although largely inhabiting rainforests, do exploit a wide range of habitats and vegetation. The combined data suggest that the site was dominated by closed-canopy rainforest but with small patches of more open canopy vegetation available in the vicinity of the site.

## 5. Conclusion

Integration of existing historical, speleological, and geological evidence suggests that the Lida Ajer fossils were all most likely deposited during MIS 4 (76–59 ka, [37]); however, suboptimal chronological resolution and dating complexities in the cave means we cannot unambiguously reconstruct the sequence of events for the formation of the cave deposits. We outline the most parsimonious sequence based on all currently available data. The earliest deposits in the cave are represented by speleothem growth during MIS 7 (224–191 ka, [37]) and 5 (132–76 ka, [37]). Subsequently, during MIS 5 the cave was filled with clastic sediments in low energy environments and initially without faunal remains. Sediment deposition in MIS 4 filled the sinkhole passages and the lower sections of the main fossil chamber with fossil-rich muds, probably under alternating high and low energy flows. This unit is capped with volcanoclastic sediments. The next unit contains mud-dominated layers with the top 2 m of these beds cemented by speleothem, which then formed a capping flowstone over the deposits. During the early Holocene, stalactites connected with the flowstone forming the pillars still present at the site today. Dissolution of the top breccia layer completed the speleological evolution of the deposits sometime before Dubois began his excavations in 1888. The vertebrate taxa inhabited an environment very similar to today, although some differences in the way niche space was occupied is likely. The human presence represented by the dental remains recovered by Dubois from Lida Ajer were therefore likely to have occupied an environment that included strong components of closed-canopy rainforest environment during MIS 4. Direct dating of the human teeth and stable isotope analyses will be required to determine exactly where in the Lida Ajer sequence they probably came from and exactly which environments they exploited.

## Data Availability

All new data are provided in full in the electronic supplementary material [45].
